# Preliminary Characterization of Mitochondrial Genome of *Melipona scutellaris*, a Brazilian Stingless Bee

**DOI:** 10.1155/2014/927546

**Published:** 2014-06-16

**Authors:** Manuella Souza Silverio, Vinícius de Rezende Rodovalho, Ana Maria Bonetti, Guilherme Corrêa de Oliveira, Sara Cuadros-Orellana, Carlos Ueira-Vieira, Anderson Rodrigues dos Santos

**Affiliations:** ^1^Universidade Federal de Uberlândia, Instituto de Genética e Bioquímica, Avenida Pará 1720, Campus Umuarama, Bloco 2E, Sala 246, 2° Piso, 38400-902 Uberlândia, MG, Brazil; ^2^René Rachou Research Center-Fiocruz, Molecular and Cellular Pathology Laboratory, Avenida Augusto de Lima 1715, Barro Preto, 30190-002 Belo Horizonte, MG, Brazil; ^3^Universidade Federal de Uberlândia, Faculdade de Computação, Avenida João Naves de Ávila 2121, Campus Santa Mônica, Bloco 1B, Sala 148, 38400-902 Uberlândia, MG, Brazil

## Abstract

Bees are manufacturers of relevant economical products and have a pollinator role fundamental to ecosystems. Traditionally, studies focused on the genus *Melipona* have been mostly based on behavioral, and social organization and ecological aspects. Only recently the evolutionary history of this genus has been assessed using molecular markers, including mitochondrial genes. Even though these studies have shed light on the evolutionary history of the *Melipona* genus, a more accurate picture may emerge when full nuclear and mitochondrial genomes of *Melipona* species become available. Here we present the assembly, annotation, and characterization of a draft mitochondrial genome of the Brazilian stingless bee *Melipona scutellaris* using *Melipona bicolor* as a reference organism. Using Illumina MiSeq data, we achieved the annotation of all protein coding genes, as well as the genes for the two ribosomal subunits (16S and 12S) and transfer RNA genes as well. Using the COI sequence as a DNA barcode, we found that *M. cramptoni* is the closest species to *M. scutellaris*.

## 1. Introduction

*Melipona scutellaris*, popularly known as* uruçu* bee, is a stingless bee species profusely found from Bahia to Pernambuco Brazilian states. They are present in urban and rural environments and their pollinator role is pivotal to the ecosystems in which they live [1, 2].

The genus* Melipona* has long been target of ecological, genetic, and especially behavioral and pollination studies. Existing molecular phylogenetic studies have shown that the* Melipona* genus clusters with other neotropical Meliponini. Furthermore,* Melipona* spp. form a well-supported monophyletic group, as determined by recent studies. Stingless bees, especially, compose an ancient group, distributed worldwide around tropics, making them important species for phylogenetic relationships studies. Thus, the elucidation of social behavioral evolution relies on a better understanding of phylogenetic relationships for eusocial insects [3, 4]. For this reason, there is an increasing interest on getting more accurate phylogenetic reconstructions, based on molecular aspects.

Phylogenetic studies have explored nuclear ribosomal genes. Since 18S and 28S subunits are short genes they are therefore easily amplified and sequenced. Besides, such genes are located in very informative regions, where conserved or variable sequences may enlighten phylogenetic relations between species [5, 6]. The mitochondrial genome has also been included in such studies, since its potential for providing evolutionary information was recognized. Metazoan mitogenomes are about 16 kb long and contain 37 genes: 22 tRNAs, 2 rRNAs (16S and 12S subunits), and 13 oxidative phosphorylation proteins—7 from complex I (ND1, ND2, ND3, ND4, ND4L, ND5, and ND6), one from complex III (cytochrome B), three from complex IV (COI, COII, and COIII), and two from complex V (ATPase6 and ATPase8) [7].

The tRNA genes are embedded in variable regions. Through evolution, these regions underwent rearrangements more often than protein coding regions. Therefore, tRNA order is a tool for comparative phylogenetic analysis between species [8]. Additionally, the high copy number per cell, low recombination rate, high mutational rate, and dominantly maternal heritance have made the mitochondrial genome a powerful tool for evolutionary studies.

DNA barcoding, or taxon identification using a standardized genomic region, was initially used in the study of animal specimens [9] and later for a wider diversity of organisms. DNA barcoding based on the cytochrome c oxidase subunit 1 (COI) gene has since then become a widely accepted molecular marker for species identification. This 650 bp long sequence is a simple and reliable tool for metazoan species identification, indicating their molecular divergences or similarities [4]. Being such a short sequence, it is useful for robust phylogeny analysis and important for tracking and measuring ecosystems biodiversity, what has lately been referred to as* metabarcoding* [10].

Other genes may be employed for DNA barcoding, but, especially among Arthropoda, the interest on COI over other genes is mainly due to its well conserved and single copy sequence, which makes the amplification by PCR reaction easier, allowing the usage of a small set of primers. Still, COI sequence presents faster substitution rates than nuclear genes and its variations are remarkably more interspecific than intraspecific [10, 11].

DNA barcoding applications for insects have been very successful [11]. This technique allows species identification in different life stages (eggs, larvae, nymphs, and pupae), when morphologic characteristics are not easily identified [12, 13]. Besides, it makes species identification possible with only tissues or fragmented parts of the insect [14]. Further, the importance of phylogenetic relationship analysis relies on understanding ecosystems biodiversity, taking into account that insects are important at pollination, decomposition, pest control, and even disease vectors [10].

The present study characterized the draft of* M. scutellaris* mitogenome annotation according to its gene order, gene conservation, and taxonomic characterization by DNA barcoding.

## 2. Material and Methods 

### 2.1. Biological Material

Total DNA was extracted from a pool of five male individuals from the Meliponary UFU at* Universidade Federal de Uberlândia*, campus Umuarama (S 180 55′/W 450 17′). DNA extraction was performed with CTAB buffer, which consists of 2% (w/v) CTAB diluted in 100 mM Tris-HCl, 20 mM EDTA, and 1.4 M NaCl. Immediately before maceration, 0.2% (v/v) *β*-mercaptoethanol was added; 150 *μ*L of CTAB buffer was added and maceration was performed manually with a pestle. Then 350 *μ*L of CTAB buffer and 5 *μ*L of RNase solution (100 mg/mL) were added, following incubation at 37°C for 1 hour. Later, 5 *μ*L of proteinase K solution (20 mg/mL) was added with additional incubation at 50°C for 1 hour. For homogenate extraction, addition of 240 *μ*L of Phenol/Chloroform/Isoamyl alcohol (25 : 24 : 1) and then centrifugation at 12,000 ×g for 10 minutes. Supernatant was transferred for a new tube and DNA was precipitated with 500 *μ*L of absolute ethanol, following centrifugation at 12,000 ×g for 15 minutes. Ethanol 70% was used for pellet washing, with a volume of 500 *μ*L and centrifugation at 12,000 ×g for 3 minutes. This step was repeated [15]. Pellet was dried at room temperature overnight and eluted into 100 *μ*L of MiliQ water.

### 2.2. Mitochondrial Genome Sequencing

The total genome sequencing of* M. scutellaris* was performed at the René Rachou Research Center, Fiocruz Minas (Belo Horizonte, MG) using an Illumina platform (MiSeq) and a paired-end strategy. The library was constructed with the Nextera XT DNA Sample Preparation Kit, following the manufacturer's instructions. Fragments of 404 bp long were carried out for sequencing. The average read length was 250 bp and the final throughput was 8.4 Gbp.

### 2.3. Genome Assembly

In order to achieve more reliable and accurate results, two kinds of assembly software were employed: SOAPdenovo2 [16] and Velvet [17]. The first* M. scutellaris* mitogenome created in this work was made using contigs generated by the SOAPdenovo2 software with varying kmer parameter between 23 and 127.

The assembly by Velvet generated assemblies for different kmer values: 31, 41, 51, 61, 71, 81, 91, or 99. The MuMmer Package 3.0 [18] aligned the contigs from each pair of assemblies with the reference sequence and show-tiling was used to complete the scaffolding of the set of contigs. Combinations of the following set of parameters were used in show-tiling: -i: 50, 70, or 90; -V: 0. 5 or 10; -v: 10, 50, or 90; -c: included or not.

The generated sequences were filtered according to the following conditions: (i) sequence size between 14,000 and 17,000 base pairs; (ii) AT content greater than or equal to 84%; (iii) maximum gap tolerance of 100 nucleotides.

**Figure 1:**
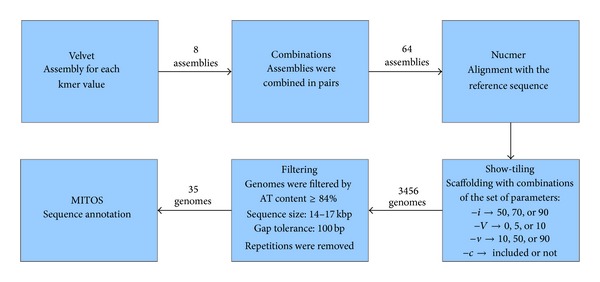
Velvet genome assembly, parameters adopted and MITOS annotation.

The remaining sequences were submitted to MITOS [19], for functional annotation. Steps for Velvet assembly and subsequent annotation with MITOS are schemed in Figure 1. The reference mitogenome of* M. bicolor* was also submitted in order to provide a more reliable basis for further analysis. The data from annotations were used for analysis of gene order and similarity to reference sequence.

The genes from the reference genome of* M. bicolor* are listed in the first column of Table 1. Relatively to each gene, the adjacent genes were analyzed (upstream and downstream) according to their frequencies (occurrence: “OCR” in Table 1). The annotated genes from* M. scutellaris* were locally aligned against the reference mitogenome, highlighting identity and* E*-value. Transfer RNAs secondary structures were predicted using tRNASCAN-SE software [20].

## 3. Results and Discussion

In total, 36 assemblies were performed, generating 36 annotations which allowed inferring that all protein-coding genes and tRNA genes were annotated. Using all annotations, the gene order analysis revealed synteny between* M. scutellaris* mitogenome and the reference genome. Ribosomal genes identities were also analyzed (Table 1), as well as the conservation of secondary structure for tRNA.

### 3.1. Organization and Partial Characterization of* M. scutellaris* Mitogenome

In Table 1, the “reference gene” column corresponds to the mitochondrial gene order of* M. bicolor*. Considering the genes that are found in the reference genome, column “OCR” (second column) shows how many times each gene is annotated for* M. scutellaris*, within the 36 annotations. “Upstream gene” and “downstream gene” columns refer to which genes are found in those respective positions, relative to the reference gene. Finally, “OCR” columns (fifth and sixth columns) mean how many times a gene is found in such position, relative to another gene, also considering the total of 36 annotations.

**Figure 2:**
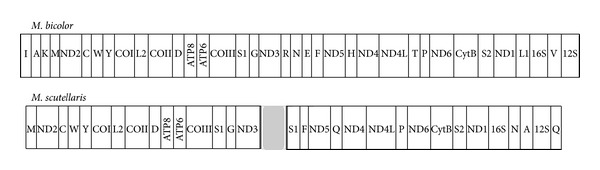
Comparison between* M. bicolor'*s mitogenome and gene organization achieved for* M. scutellaris.* Genes whose position is not yet validated were omitted (in gray).

*M. scutellaris* mitogenome shows an overall high synteny when compared to* M. bicolor* mitogenome.  Figure 2 is an illustrative scheme which gathers the annotated genes under the most frequent order, among all 36 assemblies. Although all genes were found, they are not all present in the following scheme, as long as some few ones must yet have their position validated.

**Figure 3:**
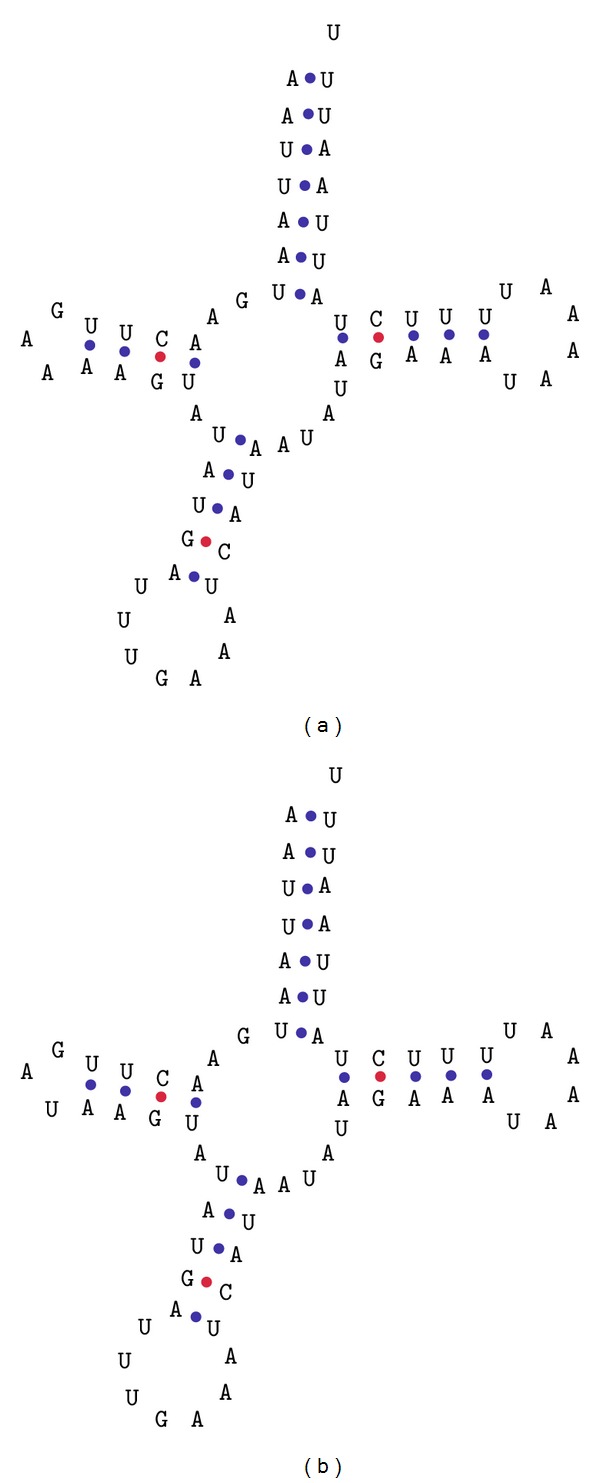
Comparison of secondary structure predictions for tRNAS2 genes of* Melipona scutellaris* (a) and* Melipona bicolor* (b).

Regarding tRNA genes, aligning the annotated genes against the reference genome did not generate considerable identity values for all cases. However, submitting the* M. scutellaris* sequences to tRNAScan-SE, it was possible to infer that the tRNA secondary structures of* M. scutellaris* are viable and well conserved. An example is displayed in Figure 3.

In Table 1 it is possible to notice that in some cases there is more than one gene in the upstream and/or downstream position, providing different possibilities of gene organization. Such genes are tRNAA, tRNAK, tRNAG, tRNAR, tRNAT, and tRNAL1.

The coding-protein genes for* M. scutellaris* are syntenic to their homologous in* M. bicolor* mitogenome. Some tRNA genes are also syntenic, namely, tRNAM, tRNAW, tRNAY, tRNAL2, tRNAD, tRNAF, tRNAP, and tRNAS2. However, it is also possible to infer that some tRNA genes have underwent rearrangement, which are tRNAV, tRNAS1, tRNAN, and tRNAA. Some other tRNA genes must yet have their position validated, since their occurrence (OCR) is very low compared to the average, such as tRNAI, tRNAG, tRNAE, and tRNAL1 (not shown in Figure 2). It is known that tRNAs order is a particular feature of each insect species [8], and tRNAs distribution, copy number, and codon usage patterns are especially important in evolutionary studies [21].

Our results provided evidences of possible duplication of two genes in* M. scutellaris* mitogenome in comparison to* M. bicolor*. In Table 1 it is possible to see the occurrences for tRNAQ downstream to ND5 and 12S genes: 35/36 and 29/36, respectively. Still, tRNAA has also high occurrences for being upstream to 12S (30/36), composing the following cluster: 16S_tRNAN_tRNAA_12S. But there is also a high occurrence for being downstream to tRNAT (27/36), whose position is not yet clear, although it is already unlikely to be part of the cluster mentioned above.

A single gene found in* M. bicolor* mitogenome was not found in* M. scutellaris*, which is tRNAH. On the other hand, gene tRNAX was annotated, suggesting a tRNA that is not identified, requiring further validation.

Moreover, submitting the mitogenome sequence of* M. bicolor* to MITOS, as a support for further analysis, a gene that is not found in the currently available sequence was annotated, namely, tRNAC. This gene was also annotated in* M. scutellaris* mitogenome, in the same position. Such synteny suggests that tRNAC gene may be present in both genomes and this information provided by MITOS may have been omitted by other kinds of annotation software.

Concerning the protein-coding regions, they are probably not yet complete. Genes' lengths are not all as the expected, preventing a conclusion about the codon usage in* M. scutellaris'* mitogenome.

Finally, comparing both assemblies, N50 values adopted were 343 for SOAPdenovo2 and 722-362 for Velvet. The longer scaffolds achieved had close lengths, which are 15580 and 15206, for SOAPdenovo2 and Velvet, respectively. In general, all assemblies generated scaffolds around 14000–15580 bp long. However, despite the good quality of the data (phred > 30), it was not possible to achieve any significant scaffold in a first approach. For this reason, the present methodology was adopted in order to reach the evidences of genes' presence. As discussed above, all protein-coding sequences were annotated, as well as transfer RNA-coding and ribosomal subunits genes. It is assumed that using a single library of inserts 400 bp long may have influences over the results under the expected, requiring a complementary library for subsequent analyses.

### 3.2. DNA Barcoding for Taxonomic Identification

The cytochrome c oxidase subunit I gene (COI) sequence annotated in this study was submitted to BOLD Systems database [22] for taxonomic analysis. It was validated as belonging to the* Melipona* genus with 100% probability.

*Melipona cramptoni* had the highest identity (98.99%) to* M. scutellaris* COI sequence. Other closest hits were* Melipona rufiventris* (98.43%) and* Melipona eburnea* (98.40%). The reference organism,* M. bicolor*, was ranked at 97th position, with 95.2% identity. This observation highlights the limitations of using* M. bicolor* as a reference for assembly.

A higher identity between* M. scutellaris* and* M. rufiventris* was already expected. Rocha et al. [23] have demonstrated by cytogenetic studies that among* Melipona* species there are different heterochromatin content and distribution patterns. Two groups may be distinguished for such characteristics that may be considered for evolutionary analysis as well.

Rocha et al. [23] established* M. bicolor* as belonging to Group I, which comprises species with less than 50% of heterochromatin. Species belonging to such group display heterochromatin content concentrated in one portion of the chromosome. On the other hand, Group II is composed of species with heterochromatin content higher than 50%, spread along the chromosome extension. By cytogenetic studies,* M. scutellaris* and* M. rufiventris* were classified as being part of Group II. Therefore, DNA barcoding result revealing a closer relation between* M. scutellaris* and* M. rufiventris* rather than* M. bicolor* confirms cytogenetic studies. However, the close relation between* M. scutellaris* and* M. cramptoni* has not yet been investigated.

## 4. Conclusion

The results generated to the mitogenome of* M. scutellaris* in this study suggest a conserved character in comparison to* M. bicolor*. Protein coding genes order especially appears to be well conserved. Some tRNA genes underwent rearrangement, but a conservation pattern could also be analyzed as well.

Through taxonomic identification search on BOLD Systems database it was possible to assume that* M. cramptoni* is the closest species to* M. scutellaris* (98.99%), although* M. bicolor* is also found among closely related species (95.2% identity). The second closest species to* M. scutellaris* found at BOLD database is* M. rufiventris*, what is confirmed by cytogenetic studies.

## Figures and Tables

**Table 1:** Annotated genes in *M. scutellaris *mitogenome, upstream/downstream relations, and similarity analysis.

Reference gene		Upstream gene		Downstream gene		Similarity to reference sequence		
Name	OCR∗	Name	OCR∗	Name	OCR∗	Blast algorithm	Best identity	*E-*value
trnI	2	trnA	2	trnQ	2	blastn	—	—

trnA	34	trnT trnN	27 25	rrnS	30	blastn	92.06	2.00*E − *021

trnK	16	***trnQ*** **	5	trnE trnM	6 4	blastn	93.62	2.00*E − *016

trnM	28	***trnX*** **	22	nad2	27	blastn	95.71	2.00*E − *028

nad2	36	trnM	27	trnC	32	tblastx	96.43	2.00*E − *018

trnC	32	nad2	32	trnW	30	blastn	—	—

trnW	32	trnC	30	trnY	25	blastn	96.92	6.00*E − *028

trnY	26	trnW	25	cox1	25	blastn	—	—

cox1	36	trnY	25	trnL2	20	tblastx	98.33	2.00*E − *099

trnL2	20	cox1	20	cox2	20	blastn	—	—

cox2	36	tnrL2	20	tnrD	32	tblastx	96.55	3.00*E − *063

trnD	32	cox2	32	atp8	31	blastn	—	—

atp8	35	trnD	31	atp6	34	tblastx	93.75	2.00*E − *005

atp6	36	atp8	34	cox3	36	tblastx	94.44	1.00*E − *009

cox3	36	atp6	36	trnV	17	tblastx	94.12	4.00*E − *027

trnS1	27	trnR nad3	12 10	trnF	19	blastn	96.88	2.00*E − *027

trnG	3	cox3	3	nad3 atp8	2 1	blastn	—	—

nad3	36	trnV cox3	17 14	trnR	24	tblastx	95.00	7.00*E − *006

trnR	24	nad3	24	trnS1 trnN	12 9	blastn	—	—

trnN	34	rrnL	25	trnA	25	blastn	96.72	9.00*E − *026

trnE	12	trnK	6	trnF	7	blastn	—	—

trnF	28	trnS1	19	nad5	15	blastn	—	—

nad5	36	trnF	15	***trnQ*** **	35	tblastx	95.83	1.00*E − *013

trnH	0						—	—

nad4	36	***trnQ*** **	35	nad4l	28	tblastx	95.00	1.00*E − *028

nad4l	33	nad4	28	trnP	26	tblastx	94.74	0.13

trnT	32	nad4l	9	trnA	27	blastn	—	—
		***trnQ*** **	7					

trnP	35	nad4l	26	nad6	35	blastn	—	—

nad6	35	trnP	36	cob	35	tblastx	94.12	4.00*E − *022

cob	36	nad6	35	trnS2	35	tblastx	95.65	1.00*E − *011

trnS2	35	cob	35	nad1	35	blastn	100.00	7.00*E − *032

nad1	36	trnS2	35	rrnL	34	tblastx	92.86	5.00*E − *021

trnL1	2	trnY nad1	1 1	rrnL trnL2	1 1	blastn	—	—

rrnL	36	nad1	34	trnN	25	blastn	95.26	3.00*E − *095

trnV	23	cox3	17	nad3	17	blastn	—	—

rrnS	36	trnA	30	tnrQ	29	blastn	95.08	4.00*E − *024

*OCR: occurrences in different assemblies.

∗∗Genes not found in *M. bicolor *mitogenome.
